# Editorial

**Published:** 1860-05

**Authors:** 


### EDITORIAL.

### CHICAGO MEDICAL JOURNAL AND THE STATE MEDICAL SOCIETY.

"THE ILLINOIS STATE MEDICAL SOCIETY AND THE NATIONAL ASSOCIATION.—At the last meeting of the Illinois State Medical Society a movement was made, looking to *reform* in the manner of electing the President of the National Association. A resolution relating to this matter was introduced, as was understood, at the instance of Dr. N. S. Davis, but did not pass. At present the Association has the right of choosing its officers from any part of the Union, although for obvious and proper reasons the Presidents have most frequently been taken from the State in which the meeting was held. Dr. Davis proposes to make a rule prohibiting such choice, and disfranchising the members of the profession in the particular State or city in which the meeting may be held, and urges his friends to rally and put it through.

This is an ingenious move. In the first place it might make his chances of election better at New Haven than they were at Washington and at Louisville, where he did not succeed. Secondly, if the Association should ever meet in this State it would prevent the election of any other person. If this resolution passes, the next move may be to invite the Association to Chicago, otherwise not.

It is melancholy to see so much attention paid to an unimportant matter, to the neglect of the scientific interest of the State and National Societies. But it is in accordance with the peculiar system of elevating the profession advocated in a certain set just at present.

For ourselves, we cannot but express a hope that our friends will, whenever consistent with their interests, attend these meetings, and that they will take pains to arrange, for the purpose of being communicated, any valuable facts or observations which may have occurred to them during the year. Thus interest may be added to the meetings, and value to the transactions."

The above taken from the *Chicago Medical Journal*, for April, 1860, makes the third time that the senior editor of that periodical has called the attention of his readers to the same subject, and each time repeating the same misrepresentations. And yet he carefully abstains from publishing the

*resolutions* alluded to, in the hope that a majority of his readers will thereby be prevented from detecting his mistatements. In the above article, Doctor Brainard, either directly expresses or plainly implies *three deliberate falsehoods*.

No. 1. " A resolution relating to this matter was introduced, as was understood, at the instance of Dr. N. S. Davis, but did not pass." Instead of originating with me, or being introduced "*at my instance*," it is well known that the resolutions originated with members of the Esculapian Society in the southern part of the State, and were introduced by Dr. T. D. Washburn, of Hillsboro, with whom I had never exchanged a word on the subject. Sometime previous to the last meeting of the State Society, I received a letter from Dr. D. W. Stormont, of Grandview, enclosing the preamble and resolutions in relation to the practice of electing the President of the American Medical Association *exclusively* from the city where the meeting was held, and asking my opinion of the propriety of having them presented to the State Society when it should meet. I wrote to Dr. Stormont, in reply, that the sentiments contained in the resolutions were correct, and suggested some verbal alterations in them; but also expressed the wish, that so far as myself was concerned, he would *not* have the subject brought before the State Society. The reason I gave was, that certain *jealous* individuals would immediately attribute the whole movement to me, and set up the pretense that it was in some way designed to favor my own election to the Presidency of the Association. From that time until they were introduced by Dr. Washburn, I neither saw nor heard anything more of the resolutions. Of the correctness of my *prediction* to Dr. Stormont, the members of the profession can now judge.

No. 2. " Dr. Davis proposes to make a rule *prohibiting* such choice, and *disfranchising* the members of the profession in the particular *State or city* in which the meeting may be held." Now read the resolution exactly as it was offered to the State Society and published in the Transactions, and see if a more bold and unblushing falsehood was ever penned?

"Therefore, *Resolved*, That in the opinion of this Society, *all the officers* of the Association should be *selected strictly with*

*reference to merit*, and *without any regard to their place of residence.*" Having thus declared in the first resolution that the officers of the Association should be selected on the principle of MERIT alone, and without any regard to their residence, the mover, in the second resolution, directly condemns the practice, hitherto generally followed, of selecting the President "exclusively" from the profession of the city in which the annual meeting is held, and thereby leaves them simply on a perfect equality with the profession of every other city and State in the Union. The resolutions, instead of disfranchising, or in any way restricting the rights of the profession in any particular locality, were founded on the broadest principle of professional equality, and were designed directly to condemn a practice which had practically given to a very small portion of the profession a MONOPALY of the highest office in its gift. All this, the senior editor of the Chicago Medical Journal very well knew, for he had the printed resolutions directly at his hand when he wrote the above article. Can the profession place a shadow of confidence in any statement that may be made by a man who thus deliberately falsifies the doings of the State Society of which he is a member?

No. 3. "In the first place it might make *his* chances of election better at New Haven than they were at Washington and at Louisville, where he did not *succeed.*" This paragraph plainly implies that at the meetings in Washington and Louisville, I was a candidate for the office of President of the Association, and *tried* to get the election; which is entirely incorrect. It is true that at Washington, it was said the *local* delegates from the profession there, would not agree on a candidate, and consequently that the name of no one in the District of Columbia would be presented to the nominating committee. On the supposition that such would be the case, several personal friends were kind enough to say to me that they would advocate my election to that office. But when the Nominating Committee met, the representative from the City of Washington was prepared to recommend a local candidate, and in accordance with previous custom he was nominated by the Committee. I neither solicited nor declined a vote on the subject. At Louisville, I am not aware that my name was ever mentioned in connection with the office of President by any one. And if it will afford the senior editor of the *Chicago Medical Journal* any relief, I will hereby give him formal notice, that in case the American Medical Association will hold its meeting for 1861 in Chicago, I will certainly not be a candidate for the office of President, nor accept it if tendered to me by the Association.

With the last paragraph, in the article quoted from the *Chicago Medical Journal*, I cordially agree; and as the senior editor has furnished but ONE formal contribution to the Transactions of the American Medical Association during the THIRTEEN years of its existence; and ONE to the Transactions of our State Society during a like period, I hope he will set the example which he urges upon others.

---

### THE CHICAGO MEDICAL JOURNAL AND DR. E. B. WOLCOTT.

The April number of the Chicago Medical Journal, contains a letter purporting to come from Milwaukee, and signed "Medicus." It is made up of some low and contemptible insinuations against the professional character of Dr. Wolcott The writer, as is usual with men when conscious of being engaged in a mean act, endeavors to hide himself behind a fictitious signature. To publish *insinuations* derogatory to the character of another, which the author neither dare to assert as facts, nor subscribe his name to, has but one paralell in meanness, and that is, robbing a neighbor's hen-roost.

---

DISCONTINUANCE. *The Peninsular and Independent*, a monthly Medical Journal, published at Detroit, has been discontinued for want of support.

### AMERICAN MEDICAL ASSOCIATION.

Before another number of the Examiner reaches its readers, the Annual Meeting of the Association will be in session at New Haven, Ct. The time appointed for the meeting is the first Tuesday in June. A more pleasant locality at that season of the year could scarcely be found on this continent, and we hope the profession of the North-West will be fully represented. A new method of business is to be inaugurated, and it is quite probable that the meeting will be more interesting and profitable than any that have preceded it. We see it intimated in one or two of our exchanges, that an attempt may be made to introduce some questions or topics calculated to stir up *sectional* feelings, having reference to the action of Southern Students in leaving Northern schools last winter.

We trust that these intimations are without the least foundation. Let politicians and divines wrangle about abstractions, until they separate on geographical lines if they will; but let the true physician know but one *section*, and that the wide world.

### TOBACCO, COFFEE AND TEA.

The Dean of Carlisle states that 33,000,000 of pounds of Tobacco were consumed in England in the year 1859; and at an expense of $40,000,000. It is estimated that the amount consumed in the United States is over 150,000,000 of pounds annually—and in the whole civilized world not less than 4,480,000,000 pounds, or 1,000,000 tons annually. It is further stated that 100,000,000 of the human race use tobacco.

The consumption of Coffee in the United States in 1859, was 251,000,000 pounds, and of Tea 36,000,000. Thus the people of the United States, in the single year 1859, paid for three articles, Tobacco, Coffee and Tea, not less than $150,000,000. If we add to this the enormous expenditure for alcoholic beverages, we shall cease to wonder why the times are hard, and the moral sensibilities of a large portion of the race blunted.

☞ We have from time to time sent out a number of extra *Examiners*, and shall continue to do so until every member of the profession in the State has seen a copy of our publication. Those who do not desire to take the *Examiner*, will confer a favor upon the editors, by returning the number received, *with address;* or signify their wish by some other mode. Otherwise, the person receiving the *Examiner* might find that he is considered a subscriber. We wish to hear from every one received either " pro or con."

---

*To the Medical Students of the United States of America.*— I will give a Premium of $250 for the ESSAY which shall be judged the best by Competent judges, on the Anatomy and Physiology of the Animal and Organic Nervous Systems. The Essays to be sent to me on or before the first of March, 1861.

I will likewise give a second premium of $250 for the best Essay on the same subject. The essays to be sent in on or before the first of March, 1862.

The Medical Students who shall be declared the Successful Competitors, will be required to declare on their word and honor, that the Essays are their own productions; and that they have not been assisted by any legally qualified Medical man.

JOHN O'REILLY, M. D.,
230 Fourth Street,
March 8th, 1860. Washington Square, New York.

---

INDIANA STATE MEDICAL SOCIETY.

The annual meeting of the Indiana State Medical Society will be held on the third Tuesday in May, at Indianapolis.

